# Reply: Association of maternal polycystic ovary syndrome or anovulatory infertility with obesity and diabetes in offspring: a population-based cohort study

**DOI:** 10.1093/humrep/deab256

**Published:** 2021-11-17

**Authors:** Xinxia Chen, Emilia Koivuaho, Terhi T Piltonen, Mika Gissler, Catharina Lavebratt

**Affiliations:** 1 School of Nursing, Cheeloo College of Medicine, Shandong University, Jinan, Shandong, China; 2 Department of Molecular Medicine and Surgery, Karolinska Institutet, Stockholm, Sweden; 3 Center for Molecular Medicine, Karolinska University Hospital, Stockholm, Sweden; 4 Department of Obstetrics and Gynecology, PEDEGO Research Unit, Medical Research Center, Oulu University Hospital, University of Oulu, Oulu, Finland; 5 Department of Information Services, Finnish Institute for Health and Welfare, Helsinki, Finland

Sir,

We would like to thank Dr Wei (2021) for the interest in our paper (Chen *et al.*, 2021) and are happy to respond.

In the letter to the editor, Dr Wei states their appreciation for the paper and they agree with most of the article content. Dr Wei correctly notices that maternal pre-pregnancy BMI is categorized into three strata: <25, 25–29 and ≥30 kg/m^2^. Dr Wei states that this is not recommended given that, according to the World Health Organization (WHO) classification (Parr *et al.*, 2010), BMI is generally divided into four categories: <18.5, 18.5–24.9, 25.0–29.9 and ≥30 kg/m^2^.

WHO has recommended classification of bodyweight including degrees of underweight and excess weight based on BMI, calculated as weight in kilograms divided by height in meters squared (kg/m^2^). However, the BMI distribution differs between ethnic groups. According to the WHO regional office for Europe, BMI falls into one of the following categories for adults over 20 years old: (i) underweight: below 18.5, (ii) normal weight: 18.5–24.9, (iii) pre-obesity: 25.0–29.9, (iv) obesity class I: 30.0–34.9, (v) obesity class II: 35.0–39.9 and (vi) obesity class III: above 40 kg/m^2^ (Office WER, 2019).

BMI groups are not distributed equally in the population. Generally, there are a few people in the underweight and obesity class III groups, and the majority of people have normal-weight, pre-obesity, or obesity class I or II. In order to have a good statistical power, the underweight group is often combined with the normal weight group, while persons with BMI over 30 kg/m^2^ are often combined in an obesity group. Specifically, in our study, there were 419 (1.7%) mothers with polycystic ovary syndrome (PCOS) who had BMI <18.5 kg/m^2^ before pregnancy. They were too few for a subgroup analysis for offspring diagnosis of obesity or diabetes. Therefore, they were combined with the normal-weight group.

Women with PCOS are more likely to be overweight or obese, and our results and others' also suggest that increased BMI at early ages might predict the development of PCOS (Brower *et al.*, 2019; Koivuaho *et al.*, 2019; He *et al.*, 2020). By focusing on the independent and interactive effects of maternal PCOS and higher BMI, our results would have implications on preventative interventions for offspring born to mothers with PCOS, particularly those with BMI over 25 kg/m^2^.

Therefore, in this study, we classified pre-pregnancy BMI into three strata in the analysis. However, we acknowledge that it would be better to categorize groups in more detail wherever the sample size is large enough. Also, there might be a pathophysiological heterogeneity in PCOS, dependent on BMI (Escobar-Morreale, 2018). For underweight or normal-weight women with PCOS, the defect in androgen synthesis is severe enough to trigger PCOS with absence of other factors such as obesity; for PCOS with overweight or obesity, a mild defect in androgen secretion is amplified by the coexistence of adiposity and/or insulin resistance to manifest as PCOS. It would be interesting to further examine offspring obesity and diabetes risks in the underweight group.

## Conflict of interest

None. 
